# Efficacy and Safety of Potassium-Competitive Acid Blockers Versus Proton Pump Inhibitors as Helicobacter pylori Eradication Therapy: A Meta-Analysis of Randomized Clinical Trials

**DOI:** 10.7759/cureus.48465

**Published:** 2023-11-07

**Authors:** Abdullah Shah, Omer Usman, Tafseer Zahra, Sandipkumar S Chaudhari, Gopi Sairam Reddy Mulaka, Rumaisa Masood, Saima Batool, Faraz Saleem

**Affiliations:** 1 Nephrology, Rehman Medical Institute, Peshawar, PAK; 2 Internal Medicine, Texas Tech University Health Sciences Center El Paso, Houston, USA; 3 Medicine, California Institute of Behavioral Neurosciences & Psychology, Fairfield, USA; 4 Cardiothoracic Surgery, University of Alabama at Birmingham, Birmingham, USA; 5 Family Medicine, University of North Dakota School of Medicine and Health Sciences, Grand Forks, USA; 6 Internal Medicine/Human Physiology, St. Martinus University Faculty of Medicine, Willemstad, CUW; 7 Internal Medicine, Services Institute of Medical Sciences, Lahore, PAK; 8 Internal Medicine, Hameed Latif Hospital, Lahore, PAK; 9 Internal Medicine, Akhtar Saeed Medical & Dental College, Lahore, PAK; 10 Internal Medicine, California Institute of Behavioral Neurosciences & Psychology, Fairfield, USA

**Keywords:** randomized clinical trials, meta-analysis, systematic review, helicobacter pylori, eradication, proton pump inhibitors, potassium-competitive acid blockers

## Abstract

*Helicobacter pylori *is a gram-negative bacterium that chronically infects the gastric epithelium. Potassium-competitive acid blockers (P-CABs) are a promising alternative, being more potent than standard proton pump inhibitors (PPIs). The meta-analysis followed Preferred Reporting Items for Systematic Reviews and Meta-Analyses (PRISMA) guidelines. Inclusion criteria were randomized controlled trials (RCTs) comparing P-CAB and PPI-based therapy, confirmed *H. pylori *infection, and measured eradication rates after at least four weeks. Subgroup analyses were conducted based on therapy type and trial location. Quality assessment used the Cochrane risk-of-bias tool, RoB 2.0, and statistical analysis was performed using ReviewManager (RevMan) 5.4 (2020; The Cochrane Collaboration, London, United Kingdom). A p-value of <0.05 is considered statistically significant. In the intention-to-treat (ITT) analysis, P-CABs demonstrated superior overall efficacy, consistently observed in the first-line treatment subgroup. However, no significant difference was found in the subgroup receiving salvage therapy. Another ITT subgroup analyzed the impact of geographical location, favoring P-CABs in the overall study population and the Japanese subgroup. However, no statistically significant differences were found in the subgroups of other countries. In the PPA, P-CABs showed superior efficacy overall, consistently seen in the first-line treatment subgroup. However, no significant difference was found in the subgroup receiving salvage eradication therapy. Another PPA subgroup analysis considered the geographical impact on eradication rates, revealing P-CABs as superior to PPIs in the overall study population and the Japanese subgroup, but not in other countries. No significant adverse event outcomes were observed. P-CAB-based triple therapy is more effective than PPI-based triple therapy as the primary treatment for *H. pylori* eradication, particularly in Japanese patients. Nevertheless, regarding salvage therapy, both treatments show comparable efficacy. Additionally, the tolerability of P-CAB-based and PPI-based triple therapy is similar, with a similar occurrence of adverse events.

## Introduction and background

*Helicobacter pylori* is a gram-negative, microaerophilic bacterium. It possesses distinct adaptations to chronically infect the luminal surface of the gastric epithelium [1]. The global prevalence of *H. pylori *infection is estimated to be around 50%, while in the United States, it is approximately 35-40%. *H. pylori* has been found to be linked to the pathogenesis of many gastroduodenal disorders [2], encompassing gastric and duodenal ulcers, gastric cancer, gastric mucosa-associated lymphoid tissue (MALT) lymphoma [3-5], and gastritis [6]. Both the Maastricht V Consensus Report [7] and the Kyoto Global Consensus Report [8] acknowledge *H. pylori*-associated gastritis as an infectious ailment, although the World Health Organization (WHO) has classed it as a carcinogenic agent [9]. The role of *H. pylori* in stomach cancer has been widely accepted for over 30 years [10], and emerging evidence suggests that eradicating *H. pylori* may decrease the incidence of gastric cancer [11-13]. The acquisition of *H. pylori* is predominantly observed during childhood [14], and its progression extends over several decades [15]. Therefore, testing young adults for *H. pylori* and eradicating it in those who test positive is a rational way to lower the risk of stomach cancer [16].

The development of a widely accepted *H. pylori *treatment remains challenging. However, all interventions aim to reduce infectious symptoms and recover mucosal integrity [17]. Primary and secondary *H. pylori* treatments with proton-pump inhibitors (PPIs) are common. However, standard treatment methods, which necessitate acid suppression and numerous antibiotics, have efficacy rates that have fallen below the deemed acceptable level of 80% worldwide [18]. The decrease in the efficiency of treatment is impacted by various factors, including antibiotic resistance, level of acid suppression, and several host and bacterial variables [19-21]. It is worth noting that in areas with clarithromycin resistance, current expert clinical practice guidelines no longer recommend normal clarithromycin-based triple therapy as the primary empirical treatment [22-24]. In 2017, the WHO declared *H. pylori* a high-priority bacterium due to the problem at hand. This designation was created to encourage scientific research and advance new approaches to treatment [25].

Potassium-competitive acid blockers (P-CABs) function by selectively and reversibly blocking the enzyme H+/K+-ATPase, effectively reducing the production of gastric acid. Their efficacy is dose-dependent, as they engage in competitive interactions with potassium ions [26]. They demonstrate a greater ability to suppress acid for a longer duration, and their impact on the CYP2C19 system is somewhat lower when compared to PPIs [27-29]. The potency of these substances exceeds that of conventional PPIs by a ratio of 350 [30,31]. Clinical research undertaken in Japan showed that P-CABs had distinct advantages over PPIs in terms of eradicating *H. pylori *[32]. Since 2015, the product has been made accessible in Japan and has also been released in a restricted number of other Asian nations [27,33-35] for the purpose of treating *H. pylori*. Recent meta-analytic studies have demonstrated the comparative advantage of therapy incorporating P-CABs in comparison to those of PPIs [36-38]. Nevertheless, the findings of this study have diverged from those of previous research [39-42]. Consequently, we have conducted a meta-analysis that explicitly included randomized controlled trials (RCTs) in order to assess the effectiveness and safety of P-CAB-based therapy for the eradication of *H. pylori*.

## Review

Methods

Our meta-analysis adhered to the guidelines set by the Preferred Reporting Items for Systematic Reviews and Meta-Analyses (PRISMA) [43].

Search Strategy

A systematic search was conducted on the Cochrane Library, Embase, Scopus, PubMed, and Google Scholar from inception until September 20, 2023, for RCTs that evaluated the eradication rate of *H. pylori.* The following key terms and words, or their equivalents, were utilized: "potassium-competitive acid blocker," "vonoprazan," "takecab," "TAK438," "Helicobacter pylori," "H. pylori," "Hp," and "Proton pump inhibitors," in conjunction with the Boolean operators "AND" and "OR." There were no restrictions imposed on time or language. Details of the search strategy employed can be found in Table 1.

**Table 1:** Search Strategy Table

Database	Query	Search details	Results
PubMed	(potassium-competitive acid blocker OR vonoprazan OR takecab OR TAK438) AND (Helicobacter pylori OR H. pylori OR Hp) AND (Proton pump inhibitors)	(("potassium-competitive"[All Fields] AND ("acids"[MeSH Terms] OR "acids"[All Fields] OR "acid"[All Fields]) AND ("blocker"[All Fields] OR "blocker s"[All Fields] OR "blockers"[All Fields])) OR ("1 5 2 fluorophenyl 1 pyridin 3 ylsulfonyl 1h pyrrol 3 yl n methylmethanamine"[Supplementary Concept] OR "1 5 2 fluorophenyl 1 pyridin 3 ylsulfonyl 1h pyrrol 3 yl n methylmethanamine"[All Fields] OR "vonoprazan"[All Fields]) OR "takecab"[All Fields] OR ("1 5 2 fluorophenyl 1 pyridin 3 ylsulfonyl 1h pyrrol 3 yl n methylmethanamine"[Supplementary Concept] OR "1 5 2 fluorophenyl 1 pyridin 3 ylsulfonyl 1h pyrrol 3 yl n methylmethanamine"[All Fields] OR "tak438"[All Fields])) AND ("helicobacter pylori"[MeSH Terms] OR ("helicobacter"[All Fields] AND "pylori"[All Fields]) OR "helicobacter pylori"[All Fields] OR ("helicobacter pylori"[MeSH Terms] OR ("helicobacter"[All Fields] AND "pylori"[All Fields]) OR "helicobacter pylori"[All Fields] OR "h pylori"[All Fields]) OR "Hp"[All Fields]) AND ("proton pump inhibitors"[Pharmacological Action] OR "proton pump inhibitors"[MeSH Terms] OR ("proton"[All Fields] AND "pump"[All Fields] AND "inhibitors"[All Fields]) OR "proton pump inhibitors"[All Fields])	204
Embase			30
Cochrane Library			18
SCOPUS			29
Google Scholar			45

Inclusion and Exclusion Criteria

To establish the study's eligibility, two reviewers independently examined pertinent literature. A third reviewer was consulted to reach a consensus in the event of differences. Our inclusion criteria encompass RCTs that compare P-CAB and PPI-based therapy as the main treatment for *H. pylori* eradication, *H. pylori *infection confirmed (by one or more confirmatory tests), the eradication rate measured using intention-to-treat (ITT) and per-protocol (PP) analyses at least four weeks after treatment completion and confirmed *H. pylori* eradication. The criteria for exclusion were observational studies and non-RCTs, absence of pertinent data, publications with abstracts only, unpublished research, non-English language, and unmeasured eradication rate.

Data Extraction and Measures of Outcomes

The following data were extracted by two independent researchers: name of the first author, publication year, country, study period, eradication regimens, eradication rate, dropout rate, and adverse events. Risk ratios and 95%CI were calculated from this data.

The outcomes of our study depend on the comparison of the rates of *H. pylori *eradication between two cohorts, the group administered with P-CAB, and the group administered with PPI. Both the ITT and PPA methodologies were employed to evaluate the rates in question. Furthermore, subgroup analyses were undertaken based on the kind of therapy (first-line and salvage) and the location of the trial, with a specific focus on Japan and other countries. The study incorporated adverse events as an extra metric for evaluation. This meta-analysis aimed to assess significant differences in outcomes between the two groups.

Quality Assessment and Statistical Analysis

An independent researcher used the Cochrane risk-of-bias tool, RoB 2.0 to assess quality (Appendix). Forest plots and statistical analyses were conducted with ReviewManager (RevMan) 5.4 (2020; The Cochrane Collaboration, London, United Kingdom). The pooled effect size was calculated using forest plots with random or fixed effects. When I2 was less than 50%, a fixed-effects model was used; otherwise, a random-effects model was used. Publication bias was assessed using a funnel plot (Figure 2). A p-value <0.05 indicated statistical significance.

**Figure 1:**
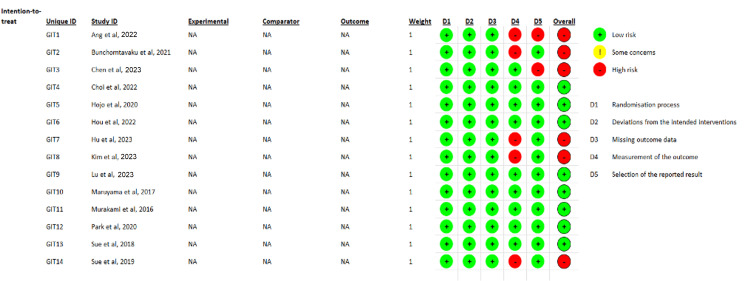
Quality Assessment References: [39,41,42,44-54]

**Figure 2:**
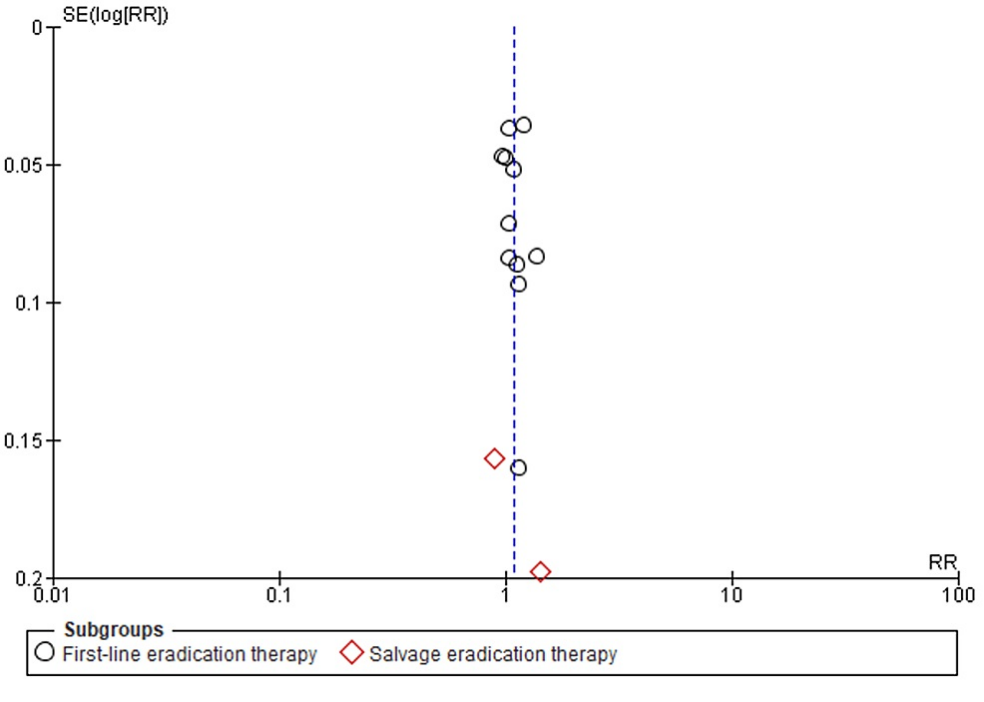
Funnel plot of comparison of P-CABs vs PPIs, with regard to ITT. P-CABs: potassium-competitive acid blockers; PPIs: proton pump inhibitors; ITT: intention-to-treat

Results

**Figure 3:**
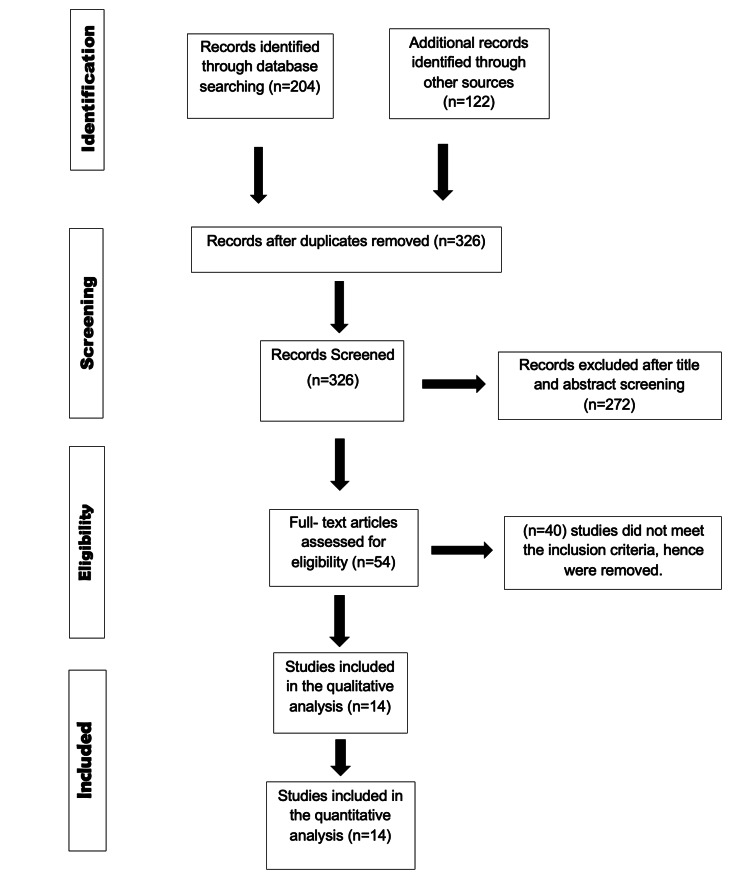
PRISMA Flow Chart PRISMA: Preferred Reporting Items for Systematic Reviews and Meta-Analyses

Figure 3 provides a visual representation of the process through which studies were chosen for inclusion in our analysis. Initially, our search yielded 326 studies from which we identified and eliminated duplicate records; 272 studies with irrelevant titles and abstracts were excluded from consideration. Fifty-four studies were chosen for further evaluation due to their relevance to the subject matter. Subsequently, an additional 40 studies were excluded. Ultimately, our final selection comprised 14 RCTs.

Table 2 shows the baseline characteristics of all included studies. The study period for the included studies ranged from 2012-2023. All of the studies were conducted in Asia, primarily in China and Japan.

**Table 2:** Baseline characteristics of the included studies. VPZ: vonoprazan; OPZ: omeprazole; RPZ: rabeprazole; ESO: esomeprazole; LPZ: lansoprazole; A: amoxicillin; C: clarithromycin; M: metronidazole; S: sitafloxcin; TPZ: tegoprazan; bd: *bis in die* (twice daily)

Author	Year	Country	Study period	Dosage of P-CAB	Dosage of antibiotics	Dosage of PPI
Hou et al. (45)	2022	China, South Korea, Taiwan, and the Philippines	2017-2019	VPZ 20 mg, bd, 14 days	A 1000 mg bd, 14 days C 500 mg bd, 14 days	LPZ 30 mg, bd, 14 days
Ang et al. (46)	2022	Singapore	2019-2021	VPZ 20 mg, bd, 7 days	A 1000 mg, bd C 500 mg, bd (For 7 or 14 days)	OPZ 20 mg, bd, 14 days ESO 20 mg, bd, 14 days RBZ 20 mg, bd, 14 days
Kim et al. (47)	2023	South Korea	2020-2021	TPZ 50 mg, bd, 14 days	Tetracycline 500 mg, qid, 14 days M 500 mg tid, 14 days	LPZ 30 mg, bd, 14 days
Hu et al. (48)	2023	China	2021-2022	VPZ 20 mg, bd, 14 days	A 1000 mg, tid or bd, 14 days M 400 mg, qid, 14 days	ESO 20 mg, bd, 14 days
Choi et al. (49)	2022	South Korea		TPZ 50 mg, bd, 7 days	A 1000 mg, bd, 7 days C 500 mg, bd, 7 days	LPZ 30 mg, bd, 7 days
Lu et al. (50)	2023	China	2021	VPZ 20 mg, bd, 10 days or 14 days	A 1000 mg, bd, 10 days or 14 days	ESO 20 mg, bd, 14 days
Chen et al. (51)	2023	China	2021-2022	VPZ 20 mg, bd, 14 days	A 1000 mg, bd, 14 days C 500mg, bd, 14 days	RPZ 10 mg, bd, 14 days
Bunchorntavakul et al. (39)	2021	Thailand	2019-2021	VPZ 20 mg, bd, 7 days	A 1000 mg bd ,C 500 mg bd, 7 days or 14 days	OPZ 20 mg, bd, 14 days
Hojo et al. (41)	2020	Japan	2015-2017	VPZ 20 mg, bd, 7 days	A 750 mg bd, 7 days M 250 mg, bd, 7 days	RPZ 10 mg, bd, 7 days
Park et al. (42)	2020	Korea	2013	YH4808 200 mg, bd, 7 days	A 1000 mg bd, 7 days C 500 mg bd, 7 days	ESO 20 mg, bd, 7 days
Murakami et al. (44)	2016	Japan	2012-2013	VPZ 20 mg, bd, 7 days	A 750 mg bd, 7 days C 200 or 400 mg, bd, 7 days	LPZ 30 mg, bd, 7 days
Maruyama et al. (52)	2017	Japan	2015-2016	VPZ 20 mg, bd, 7 days	A 750 mg bd, 7 days C 200 or 400 mg, bd, 7 days	RPZ 20 mg or LPZ 30 mg bd, 7 days
Sue et al. (53)	2019	Japan	2015-2017	VPZ 20 mg, bd, 7 days	A 750 mg, bd, 7 days S 100 mg, bd, 7 days	LPZ 30 mg, bd, 7 days ESO 20 mg, bd, 7 days RPZ 10 mg, bd, 7 days
Sue et al. (54)	2018	Japan	2015-2016	VPZ 20 mg, bd, 7 days	A 750 mg, bd, 7 days C 200, bd, 7 days	LPZ 30 mg, bd, 7 days ESO 20 mg, bd, 7 days RPZ 10 mg or 400 mg, bd, 7 days

Results of Intervention: Eradication Rate Assessed by ITT Analysis

**Figure 4:**
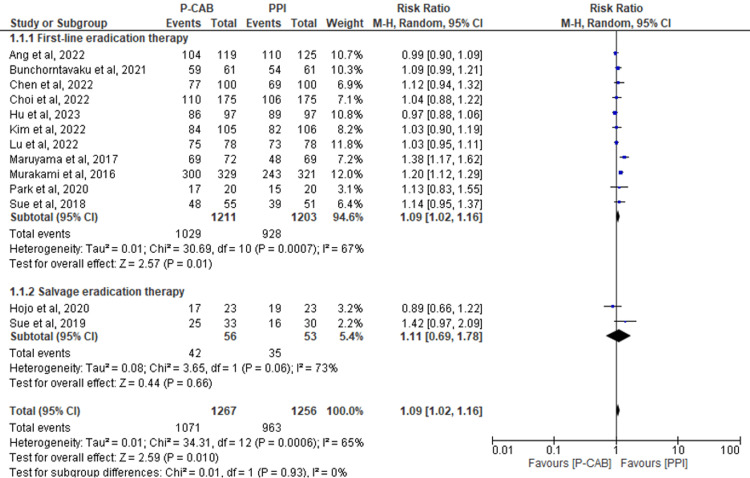
Forest plot comparing P-CABs and PPIs with regard to ITT. References: [39,41,42,44,46-54]

Subgroup analysis based on the type of therapy: A total of 13 RCTs assessed the outcome of the eradication rate by ITT analysis, revealing significant overall results favoring the P-CAB group over the PPI group (risk ratio (RR)=1.09; 95%CI: 1.02-1.16; P= 0.01; I2= 65%). The results were consistent for the subgroup of first-line eradication therapy (RR=1.09; 95%CI: 1.02-1.16; P= 0.01; I2= 67%). However, no significant differences were observed between the two groups for salvage therapy (RR=1.11; 95%CI: 0.69-1.78; P= 0.66; I2= 73%) (Figure 4).

**Figure 5:**
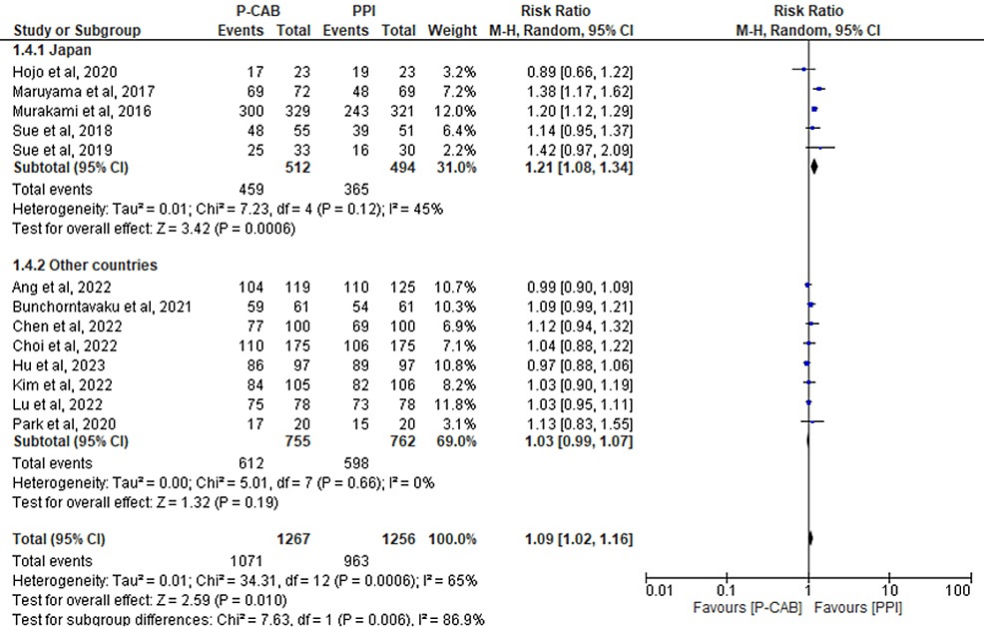
Forest plot comparing P-CABs and PPIs with regard to ITT subgroup based on countries. References: [39,41,42,44,46-54] P-CAB: potassium-competitive acid blocker; PPI: proton-pump inhibitor; ITT: intention-to-treat

Subgroup analysis based on the country in which the study was conducted: Incorporating a total of 13 RCTs that evaluated eradication rates through an ITT analysis, the findings indicated significant overall results in favor of the P-CAB group compared to the PPI group (RR=1.09; 95%CI: 1.02-1.16; P= 0.01; I2= 65%). These results remained consistent when examining the subgroup specific to Japan (RR=1.21; 95%CI: 1.08-1.34; P< 0.0006; I2= 45%). However, for the remaining countries, no significant differences were detected between the two groups (RR=1.03; 95%CI: 0.99-1.07; P= 0.19; I2= 0%) (Figure 5).

Results of Intervention: Eradication Rate Assessed by PPA

**Figure 6:**
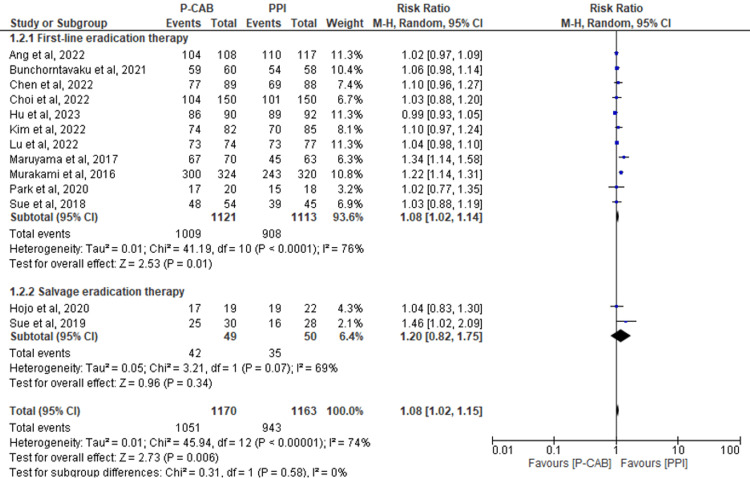
Forest plot of comparison of P-CABs vs PPIs with regard to PPA. References: [39,41,42,44,46-54] P-CAB: potassium-competitive acid blocker; PPI: proton-pump inhibitor; PPA: per-protocol analysis

Subgroup analysis based on the type of therapy: Thirteen RCTs were conducted to evaluate eradication rates using a PPA. The results demonstrated significant overall findings, with a preference for the P-CAB group over the PPI group (RR=1.08; 95%CI: 1.02-1.15; P= 0.006; I2= 74%). These results remained consistent when examining the subgroup focused on first-line eradication therapy (RR=1.08; 95%CI: 1.02-1.14; P= 0.01; I2= 76%). However, for salvage therapy (RR=1.20; 95%CI: 0.82-1.75; P= 0.34; I2= 69%), no significant differences were observed between the two groups (Figure 6).

**Figure 7:**
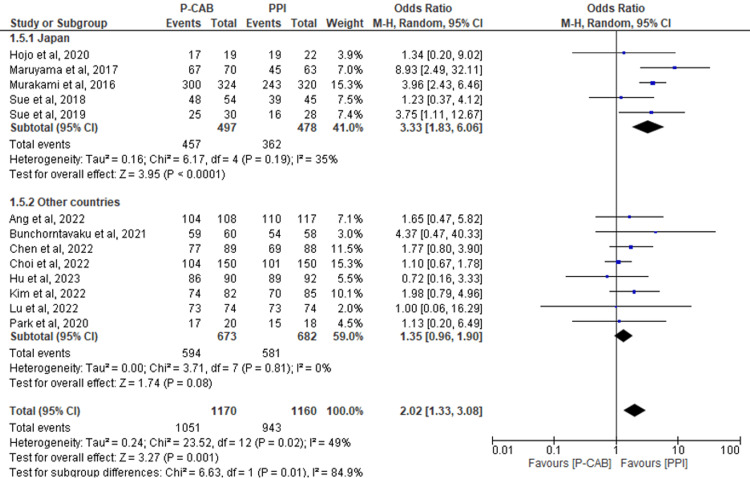
Forest plot comparing P-CABs and PPIs with regard to PPA subgroup based on countries. References: [39,41,42,44,46-54] P-CAB: potassium-competitive acid blocker; PPI: proton-pump inhibitor; PPA: per-protocol analysis

Subgroup analysis based on the country in which the study was conducted: Pooling data from a total of 13 RCTs that assessed eradication rates using a PPA, our analysis revealed significant overall results favoring the P-CAB group over the PPI group (RR=2.02; 95%CI: 1.33-3.08; P= 0.001; I2= 49%). These findings remained consistent when we analyzed the subgroup-specific to Japan (RR=3.33; 95%CI: 1.83-6.06; P< 0.0001; I2= 35%). However, for the other countries included in the study, no significant differences were identified between the two groups (RR=1.35; 95%CI: 0.96-1.90; P= 0.09; I2= 0%) (Figure 7).

Adverse Events

**Figure 8:**
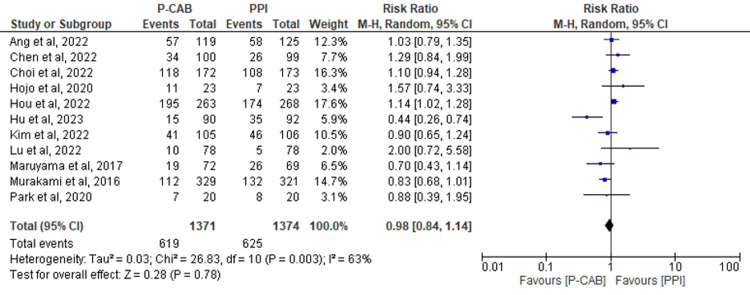
Forest plot comparing P-CABs and PPIs with regard to adverse events. References: [41,42,44-52] P-CAB: potassium-competitive acid blocker; PPI: proton-pump inhibitor

Eleven RCTs reported adverse events following the administration of P-CABs and PPIs. No significant difference was found between the two groups (RR=0.98; 95%CI: 1.14-0.93; P= 0.78; I2= 63%) (Figure 8).

Discussion

The current meta-analysis compares P-CABs to PPIs in eradicating *H. pylori *and includes both the ITT analysis and the PPA. In the ITT analysis, the overall results favored P-CABs, and the results of the subgroup first-line treatment within the ITT analysis remained consistent. However, no significant difference was found between the two groups for the subgroup of salvage eradication therapy within the ITT analysis. Another subgroup analysis was conducted within the ITT analysis to determine the effect of the country in which the study was conducted on the eradication rate, revealing P-CABs as the superior therapy over PPIs overall and within the subgroup of Japan. The subgroup of other countries did not yield significant results. In the PPA, the overall findings favored P-CABs, and this preference persisted when examining the subgroup focused on first-line treatment within the PPA. However, for the subgroup of salvage eradication therapy within the PPA, no statistically significant difference was observed between the two groups. Another subgroup analysis was conducted within the PPA to assess the impact of the study's location on the eradication rate. This analysis revealed P-CABs as the superior therapy over PPIs both in the overall study population and within the subgroup of Japan. However, the subgroup comprising other countries did not yield statistically significant results. Regarding adverse events, no significant results were obtained.

Previous meta-analyses have been conducted to evaluate the efficacy of P-CABs and PPIs in the eradication of *H. pylori*. Consistent with the findings of our study, a meta-analysis by Zhang et al. showed that P-CABs were more effective than PPIs as the first-line treatment for *H pylori* eradication (RR=1.18; 95%CI=1.10-1.28; p < 0.0001), particularly in Japanese patients [38]. However, this study only included seven RCTs, whereas our meta-analysis incorporated 14 RCTs with a larger sample size and patients from different Asian countries. Simadibrata et al. also revealed similar results, favoring the P-CABs group over PPIs in eradicating *H. pylori *(RR 1.13; 95%CI 1.04-1.22) [37]. Comparable results were shown by a meta-analysis conducted by Jung et al., revealing that the vonoprazan-based triple therapy showed superior efficacy in terms of *H. pylori* eradication compared to the PPI-based triple therapy (RR 1.19; 95%CI = 1.15-1.24) [36]. Additionally, the vonoprazan-based triple therapy showed comparable tolerability and incidence of adverse events (RR 1.02; 95%CI = 0.78-1.34). The current meta-analysis was unsuccessful in reporting significant effects of either of these medications on adverse events as well.

The effectiveness of *H. pylori* eradication is contingent upon maintaining stomach pH levels above 4.0 [55]. Vonoprazan is recognized for its capacity to sustain its acid-inhibitory activity regardless of the pH level in the gastric region, thus keeping its effectiveness in acidic conditions and preventing degradation [56]. In contrast to traditional PPIs, vonoprazan has a distinct mechanism of action that does not rely on acid activation [57]. This compound is characterized by its quick absorption in the colon, leading to efficient suppression of acid release [26]. Additionally, it should be noted that the effectiveness of vonoprazan is unaffected by meal timing, resulting in consistent medication concentrations in the bloodstream [58]. The plasma half-life of vonoprazan at a 20 mg dose is reported to be significantly longer compared to that of conventional PPIs [59]. These attributes play a significant role in achieving high eradication rates by sustaining higher pH levels.

Vonoprazan demonstrates the ability to elevate intragastric pH levels to a value exceeding 4.0 within a time frame of four hours following the initial administration in humans [59]. This creates a stable environment for preserving the effectiveness of amoxicillin and clarithromycin. Less acidity promotes bacterial growth, making them more sensitive to acid-sensitive medications like clarithromycin and amoxicillin [58]. This may provide an explanation for the observed superiority of a P-CAB-based treatment over a PPI-based regimen in patients with clarithromycin-resistant strains [44]. Furthermore, combining clarithromycin with vonoprazan leads to higher vonoprazan levels. This is attributed to clarithromycin's inhibition of the CYP3A4 enzyme, which plays a key role in metabolizing P-CAB [60].

The use of P-CABs in the treatment of *H. pylori *infection has important clinical and scientific ramifications. Clinical evidence suggests that P-CAB-based therapy may be an effective choice for the initial treatment of *H. pylori *due to its superiority, particularly in Japanese patients. This discovery might affect clinical recommendations and treatment strategies, improving patient outcomes. In terms of research, the examination of P-CABs in various geographic contexts emphasizes the significance of taking regional variations into account when creating treatment options. A customized approach to *H. pylori *eradication therapy may be made possible by further investigation into the processes underpinning P-CAB efficacy and its potential for personalized medicine methods. Studies comparing P-CABs to conventional therapies can provide a more thorough picture of the long-term effects and cost-effectiveness of P-CABs. Studies comparing the effectiveness and cost-efficiency of P-CABs to conventional treatments can also help us gain a deeper grasp of their therapeutic relevance.

The current meta-analysis demonstrates the benefits of P-CAB-based eradication therapy. However, it also has limitations. The research was exclusively conducted in Asian nations, which may have introduced selection bias due to genetic gastric pH and regional food. Second, antibiotic resistance was not examined in the study, and salvage therapy used different antibiotics. There were small sample sizes comparing P-CABs and PPIs in salvage therapy, especially in non-Japanese populations.

## Conclusions

P-CAB-based triple therapy demonstrates superior efficacy compared to PPI-based triple therapy when used as the initial treatment for *H. pylori *eradication, especially in Japanese patients. However, in the context of salvage therapy, there is no significant difference in efficacy between the two treatments. Furthermore, the tolerability of P-CAB-based and PPI-based triple therapy is similar, with a comparable incidence of adverse events.
